# Characterization of novel CD19-specific VHHs isolated from a camelid immune library by phage display

**DOI:** 10.1186/s12967-023-04524-6

**Published:** 2023-12-08

**Authors:** Mahmoud Ganji, Pooria Safarzadeh Kozani, Fatemeh Rahbarizadeh

**Affiliations:** 1 Department of Medical Biotechnology, Faculty of Medical Sciences, Tarbiat Modares University, Tehran, Iran; 2 Research and Development Center of Biotechnology, Tarbiat Modares University, Tehran, Iran

**Keywords:** CD19, Cancer immunotherapy, VHH, Nanobody, mAb, Phage display

## Abstract

**Background:**

Monoclonal antibody (mAb)-based immunotherapies have achieved promising outcomes in the treatment of immunological and oncological indications. CD19 is considered one of the most qualified antigens in the treatment of B-cell neoplasms. VHHs (nanobodies) are known for their physicochemical advantages over conventional mAbs rendering them suitable therapeutics and diagnostic tools. Herein, we aimed to isolate CD19-specific VHHs from a novel immune library using phage display.

**Methods:**

An immune VHH gene library was constructed. Using phage display and after five biopanning rounds, two monoclonal CD19-specific VHHs were isolated. The selected VHHs were expressed, purified, and characterized in terms of their affinity, specificity, sensitivity, and ability to target CD19-positive cell lines. Moreover, in silico analyses were employed for further characterization.

**Results:**

A VHH library was developed, and because the outputs of the 4^th^ biopanning round exhibited the most favorable characteristics, a panel of random VHHs was selected from them. Ultimately, two of the most favorable VHHs were selected and DNA sequenced (designated as GR37 and GR41). Precise experiments indicated that GR37 and GR41 exhibited considerable specificity, sensitivity, and affinity (1.15 × 10^7^ M^−1^ and 2.08 × 10^7^ M^−1^, respectively) to CD19. Flow cytometric analyses revealed that GR37 and GR41 could bind CD19 on the surface of cell lines expressing the antigen. Moreover, in silico experiments predicted that both VHHs target epitopes that are distinct from that targeted by the CD19-specific single-chain variable fragment (scFv) FMC63.

**Conclusion:**

The selected VHHs can be used as potential targeting tools for the development of CD19-based immunotherapeutics.

## Introduction

Over the past two decades, cancer immunotherapy has been known as a promising approach to the treatment of cancer. Approaches such as the use of monoclonal antibodies (mAbs), antibody–drug conjugates (ADCs), chimeric antigen receptor (CAR) T cells, and T-cell-redirecting bispecific antibodies (TRBAs) have completely revolutionized the face of the fight against cancer [1]. In the case of B-cell malignancies, rituximab (Rituxan^®^; which is a CD20-specific mAb approved by the US Food and Drug Administration (FDA) in 1997 for the treatment of non-Hodgkin lymphoma (NHL) and chronic lymphocytic leukemia (CLL)) has been one of the outstanding examples of the cancer immunotherapy success [2]. However, due to the lack of CD20 expression in other B-cell malignancies including multiple myeloma (MM) and B-cell acute lymphoblastic leukemia (B-ALL), CD20-based immunotherapies are not as applicable in such patients; therefore, alternative target antigens are of paramount importance [3]. Among alternative antigens (including CD19, CD22, CD37, and CD79B), CD19 seems to be the most qualified one because of its expression in B-cell lymphomas and leukemias and its absence on a wide spectrum of irrelevant healthy tissues alongside plasma cells and hematopoietic stem cells [3]. Also, it has been evident that CD19 maintains its expression even after CD20 expression loss or accentuated CD20 down-regulation [4].

The clinical success of CD19-based treatment modalities eventually led to their commercial success with blinatumomab (Blincyto^®^) being the first product FDA-approved in 2014 [5]. Blinatumomab is a TRBA used for the treatment of patients with Philadelphia chromosome-negative relapsed or refractory (R/R) B-ALL [5]. In 2020, two other anti-CD19 mAbs received FDA approval including inebilizumab (Uplizna^®^), a humanized mAb used for the treatment of adults with neuromyelitis optica spectrum disorder (NMOSD), and tafasitamab (Monjuvi^®^), which is a humanized cytolytic mAb used for the treatment of adult patients with R/R diffuse large B-cell lymphoma (DLBCL) in combination with lenalidomide (Revlimid^®^) [6, 7]. Additionally, tisagenlecleucel (Kymriah^®^) [8], brexucabtagene autoleucel (Tecartus^®^) [9], axicabtagene ciloleucel (Yescarta^®^) [10], and lisocabtagene maraleucel (Breyanzi^®^) [11] are also among CD19-redirected CAR-T therapies that have been FDA-approved for the treatment of particular subtypes of patients with CD19-positive blood-based malignancies [12]. The approval of such CD19-based immunotherapeutics highlights the therapeutic importance of this target antigen, presenting it as one of the high-profile target antigens of cancer immunotherapy [3]. In this regard, various pharmaceutical companies are currently developing different CD19-based immunotherapeutics, which are currently under clinical investigation or planned to be in the years to come.

VHHs (also known as single-domain antibody (sdAb) fragments or nanobodies), which are a particular class of antibodies that are derived from the camelid heavy-chain-only antibodies (HCAbs), have received a considerable deal of attention as potential therapeutic and diagnostic tools [13–15]. This is mainly because of the favorable properties of VHHs including their relatively small size of ~ 15 kDa (in comparison with that of a conventional single-chain variable fragment (scFv) which is ~ 30 kDa), production affordability, ease of modification, high tissue penetration rate, rapid clearance from the circulation, high solubility, and high stability index, as well as their high degree of specificity, sensitivity, and affinity [13, 16–18]. So far, VHHs have been utilized for the delivery of radioisotopes or therapeutic agents, tumor tissue imaging, and as the targeting domains of CAR construct, and they have proven to be of high value [13, 15]. The main objective of the current study was to isolate CD19-specific VHHs using the phage-display technique. An immune VHH gene library was constructed and after careful screening steps, two CD19-specific VHHs were isolated and characterized both in vitro and in silico. Ultimately, it was demonstrated that the selected VHHs are CD19-specific and that they have the potential to be utilized for the development of a wide spectrum of therapeutics such as radionuclide therapy, CAR-Ts, and nanobody-drug conjugates.

## Materials and methods

### Antibodies and antigens

Recombinant CD19 antigen protein was purchased from Abcam (Cambridge, United Kingdom) and fluorescein isothiocyanate (FITC)-conjugated anti-human CD19 antibodies (referred to as “anti-CD19 commercial antibody”) were purchased from BioLegend (San Diego, CA, United States). Furthermore, mouse horseradish peroxidase (HRP)-conjugated anti-His tag mAbs were purchased from Abcam (Cambridge, MA, United States) and HRP-conjugated anti-M13 was purchased from Sino Biological (Sino Biological, Inc., Beijing, China). Mouse anti-VHH antibodies were previously developed in our laboratory and FITC-conjugated goat anti-mouse IgG was purchased from BioLegend (San Diego, CA, United States).

### Camel immunization

To develop our CD19-specific VHH gene library, an adult healthy camel was considered for the immunization process. In detail, the camel was immunized subcutaneously five times at seven day intervals with the CD19-positive cell lines Namalwa and Raji (10^6^ cells/mL of each cell line; obtained from the Iranian Biological Resource Center, Iran) along with 2 mL of Freund’s complete adjuvant (Sigma-Aldrich, Merck KGaA, Germany) for the first round of the injections. Furthermore, according to standard immunization protocols, all subsequent boosting injections were carried out with incomplete Freund’s adjuvant (Sigma-Aldrich, Merck KGaA, Germany). Two weeks following the last round of immunization, 100 mL of non-coagulated peripheral blood sample was collected from the animal, and lymphocytes were isolated by the density gradient centrifugation method using Ficoll-Hypaque (Lymphodex, Inno-Train, Germany) as per the manufacturer’s instructions. Eventually, the collected lymphocytes were used for the preparation of the desired library as detailed in the upcoming sections. Of note, before immunization and after each round of booster injection, 1 mL of peripheral blood was collected from the animal from which serum was isolated and used for enzyme-linked immunosorbent assay (ELISA) to assess the immunization process. All of the mentioned experiments were performed in accordance with standard animal welfare regulations as approved by the *Research Ethics Committees of Tarbiat Modares University* (approval ID: IR.MODARES.REC.1400.056).

### VHH library construction

The total RNA of the obtained peripheral lymphocytes was extracted and used as the template for cDNA synthesis using oligo dT primers and Moloney Murine Leukemia Virus-derived (M-MULV) reverse transcriptase enzyme. In an attempt to only amplify the VHH encoding DNA fragments, the approach of two-step nested PCR was carried out. In detail, the first round of PCR was performed with 8 pairs of primers (Table 1) specifically designed for the amplification of the VHH DNA fragments, rather than classical VH DNA fragments, while using cDNA as template. After the completion of the first PCR round, the resultant PCR amplicons were verified in terms of length (ranging from 600 to 700 bp) by agarose gel electrophoresis, and the bands corresponding to the desired DNA fragments were subsequently extracted from the gel using an Agarose Gel DNA Extraction Kit (Roche, Mannheim, Germany). These obtained DNA fragments were considered templates for the second round of PCR. Herein, the VHH-encoding DNA fragments corresponding to framework 1 to framework 4 were amplified using 11 specifically designed pairs of primers (Table 2) that harbored the SfiI restriction enzyme sites at both ends. Eventually, the resultant PCR amplicons (ranging from 400 to 500 bp) were verified by agarose gel electrophoresis, and later on, extracted from the gel for the rest of the experiments. Next, the resultant gene fragments were enzymatically digested using the SfiI restriction enzyme (Thermo Fisher Scientific, United States) and then ligated into SfiI pre-digested pComb3XSS phagemid vectors (Addgene, Cambridge, MA, United States) in the presence of T4 DNA ligase (Thermo Fisher Scientific, United States). The resultant recombinant phagemid vectors were transformed into *Escherichia coli* (*E. coli*) strain ER2738 via electroporation (2500 V, 5 ms), as they were supplied with fresh Luria–Bertani (LB) broth media and incubated at 37 °C for 1 h (250 rpm). Next, the bacterial cells were cultured on ampicillin (100 μg/mL)-containing LB agar culture plates. Of note, the number of plaques through their serial dilution (10^–7^, 10^–8^, and 10^–9^) was used for the determination of the library size. To verify the cloning process of our VHH gene fragments into the pComb3XSS phagemid, colony PCR was performed on randomly selected colonies using specific primers (5′—AAGACAGCTATCGCGATTGCAG—3′ and 5′—GCCCCCTTATTAGCGTTTGCCATC—3′, as forward and reverse primers, respectively) [19]. In the next step, the recombinant bacteria cells were exposed to the helper phage M13KO7 (1 × 10^11^ pfu/mL) to superinfect them, and after the phages were released from the bacteria cells, they were isolated and purified using a 20% (w/v) PEG/NaCl solution (PEG 6000 purchased from Sigma-Aldrich, Merck KGaA, Germany; 2.5 M NaCl) and centrifugation at 19,000 ×*g* for 30 min at 4 °C [20].

**Table 1:** Primers used for the first round of nested PCR for VHH library construction

Pair	Designation	Sequence	References
1st	CH2-m-For-1	5′—CTGTTCCTCCTTTGGCTTCGTGTT—3′	[68]
	Bq-CH2-ca2-R	5′—GGTACGTGCTGTTGAACTGTTCC—3′	[69]
2nd	VHBACKA6	5′—GATGTGCAGCTGCAGGCGTCTGG(A\G)GGAGG—3′	[70]
	CH2FORTA4	5′—CGCCATCAAGGTACCAGTTGA—3′	
3rd	CALL001	5′—GTCCTGCTGCTCTTCTACAAGG—3′	[71]
	CALL002	5′—GGTACGTGCTGTTGAACTGTTCC—3′	
4th	CH2-m-For-1	5′—CTGTTCCTCCTTTGGCTTCGTGTT—3′	[68]
	VHH-m-Back-1	5′—TGGGTGGTCCTGGCTGCTCTT—3′	
5th	AlpVh-LD	5′—CTTGGTGGTCCTGGCTGC—3′	[72]
	CH2-R	5′—GGTACGTGCTGTTGAACTGTTCC—3′	
6th	Bq-lead-lg-F	5′—GTCCTGGCTGCTCTWYTACARGG—3′	[69]
	Bq-CH2-ca2-R	5′—GGTACGTGCTGTTGAACTGTTCC—3′	
7th	SM017	5′—CCAGCCGGCCATGGCTCAGGTGCAGCTGGTGGAGTCTGG—3′	[69]
	SM018	5′—CCAGCCGGCCATGGCTGATGTGCAGCTGGTGGAGTCTGG—3′	
8th	Bq-lead-lg-F	5′—GTCCTGGCTGCTCTWYTACARGG—3′	[69]
	VHH-m-Back-1	5′—TGGGTGGTCCTGGCTGCTCTT—3′	[68]

**Table 2:** Primers used for the second round of nested PCR for VHH library construction

Pair	Designation	Sequence	References
1st	Fr4-SfiI	5′—ACTGGCCCAGGCGGCCGAGGTGCAGCTGSWGSAKTCKG—3′	[73]
	Fr1-SfiI	5′—ACTGGCCGGCCTGGCCTGAGGAGACGGTGACCWGGGTC—3′	
2nd	VHH_For	5′—GTTATTACTCGCGGCCCAGCCGGCCATGGCCGATGTGCAGCTGCAGGAGTCTGGRGGAGG—3′	[74]
	VHH_Rev_IgG2	5′—GGTGATGGTGTTGGCCTCCCGGGCCGGCCGCTGGTTGTGGTTTTGGTGTCTT—3′	
3rd	VHH_For	5′—GTTATTACTCGCGGCCCAGCCGGCCATGGCCGATGTGCAGCTGCAGGAGTCTGGRGGAGG—3′	[74]
	VHH_Rev_IgG3	5′—GGTGATGGTGTTGGCCTCCCGGGCCGGCCGCGGAGCTGGGGTCTTCGCTGTG-3'	
4th	VHH F	5'-CTGGCCCAGGCGGCCGAGGTGCAGCTG(C/G)(A/T)G(C/G)A(G/T)TC(G/T)G-3'	[75]
	VHH R	5'-ACTGGCCGGCCTGGCCTGAGGAGACGGTGATGACC(A/T)GGGTC-3'	
5th	Ryckaert et al. 1	5'-AAAGAGAGGCCGAAGCGGCCGTGCAGCTGGTGGAGTCTG-3'	[77]
	Ryckaert et al. 2	5'-TTCGAAGGCCCCACCGGCCGAGGAGACGGTGACCTGGGT-3'	
6th	VH1	5'-CATGCCATGACTCGCGGCCCAGGCGGCCATGGCCCAGGTGCAGCTGGTGCAGTCTGG-3'	[77]
	VH1b-SfiI	5'-GCTGGATTGTTATTACTCGCGGCCCAGCCGGCCATGGCCCAGGTSMARCTGCAGSAGTCWGG-3'	[78]
7th	VH3	5'-CATGCCATGACTCGCGGCCCAGGCGGCCATGGCCGAGGTGCAGCTGGTGGAGTCTGG-3'	[77]
	VH1b-SfiI	5′—GCTGGATTGTTATTACTCGCGGCCCAGCCGGCCATGGCCCAGGTSMARCTGCAGSAGTCWGG—3′	[78]
8th	VH4	5′—CATGCCATGACTCGCGGCCCAGGCGGCCATGGCCCAGGTGCAGCTGCAGGAGTCGGG-3'	[77]
	VH1b-SfiI	5′—GCTGGATTGTTATTACTCGCGGCCCAGCCGGCCATGGCCCAGGTSMARCTGCAGSAGTCWGG—3′	[78]
9th	VH1	5′—CATGCCATGACTCGCGGCCCAGGCGGCCATGGCCCAGGTGCAGCTGGTGCAGTCTGG—3′	[77]
	VH6b-SfiI	5′—CGTGGATTGTTATTATCTGCGGCCCAGCCGGCCATGGCCGATGTGCAGCTGCAGGCGTCTGGRGGAGG—3′	[78]
10th	VH3	5′—CATGCCATGACTCGCGGCCCAGGCGGCCATGGCCGAGGTGCAGCTGGTGGAGTCTGG—3′	[77]
	VH6b-SfiI	5′—CGTGGATTGTTATTATCTGCGGCCCAGCCGGCCATGGCCGATGTGCAGCTGCAGGCGTCTGGRGGAGG—3′	[78]
11th	VH4	5′—CATGCCATGACTCGCGGCCCAGGCGGCCATGGCCCAGGTGCAGCTGCAGGAGTCGGG—3′	[77]
	VH6b-SfiI	5′—CGTGGATTGTTATTATCTGCGGCCCAGCCGGCCATGGCCGATGTGCAGCTGCAGGCGTCTGGRGGAGG—3′	[78]

### Phage library biopanning for the selection of CD19-specific VHHs

A biopanning process was carried out to select the phages from the phage library that display CD19-specific VHHs at their surface. To take this step, 96-well ELISA microplates were coated with CD19 (250 ng/well; 100 µL per well; 48 wells) or BSA (500 ng/well; 100 µL per well; 48 wells) and were later on incubated at 4 °C overnight. Further on, the excess antigen solutions were extracted from the wells, and then the wells were washed twice with phosphate-buffered saline (PBS), and next fully blocked by PBS containing 1% (w/v) skimmed milk (300 µL per well) along with 1 h of incubation at 37 °C. Next, each well was supplied with 200 μL of the phage library (containing a titer of 10^11^–10^12^ particles/mL) and later on, incubated at 37 °C for 2 h after being rinsed of any residual blocking solution. Then, the wells were washed five times with PBS containing 0.5% Tween (PBST) and twice with PBS. Next, 200 μL of elution buffer (triethylamine; TEA) was added to each well and the resulting solutions were extracted after a 10 min incubation at room temperature. Of note, Tris–HCl (1 M) (100 µL) was added to the resulting eluted phages to achieve a pH of 7.5. Moreover, the eluted phages were used to infect log-phase bacterial cells of ER2738 (1 mL of eluted phages per each 5 mL culture of log-phase bacterial cells) followed by the addition of M13KO7 helper phage (10^9^ pfu per each mL of primary culture medium). After performing the necessary incubations, a centrifugation step was carried out and the pellet was resuspended in 100 mL 2XYT culture media. Next, the necessary amounts of ampicillin (100 μg/mL) and kanamycin (50 μg/mL) were added to the medium, and then it was incubated at 37 °C overnight (250 rpm). In the following step, centrifugation was performed and then a PEG/NaCl solution (as previously detailed) was added to the supernatant for phage precipitation. The phages were pelleted by centrifugation at 19,000 ×*g* for 30 min at 4 °C, and then the pellet was resuspended in TBS, after which filtration was carried out using 0.45 μm filters. The outputs of each round of biopanning were used as the inputs for the next round. Collectively, five rounds of consecutive biopanning were carried out with each round having stricter selection conditions than the previous one resulting in gradual enrichment of the CD19-specific phage particles. Of note, 250, 225, 200, 175, 150 ng/well CD19 (100 µL per well) were coated in the ELISA plate wells for the 1st, 2nd, 3rd, 4th, and 5th round of biopanning, respectively, with each successive biopanning round having two more washing steps (once with PBST and once with PBS) than the previous one. Also, the outcomes of each round of biopanning were verified by performing colony PCR on randomly selected colonies.

### Polyclonal phage ELISA

The outputs of each round of biopanning were used for the infection of log-phase ER2738 bacterial cells. Next, M13KO7 helper phages were added to the bacterial cells. A phage enrichment step was carried out as discussed in the previous section, and the phage particles were resuspended in TBS and subsequently filtered via 0.45 μm filters. The phage outputs from each round of biopanning were used for assessing the binding capacity of that biopanning round to CD19. In brief, 200 ng/well of BSA or CD19 (100 µL per well) were coated in ELISA plates and then incubated at 4 °C overnight. Next, the coated wells were blocked by PBS containing 1% (w/v) skimmed milk by being incubated at 37 °C for 1 h. In the next step, the wells were washed five times with PBS, and then phage particles (10^10^ particles per well) were added to the CD19- or BSA-coated wells as the ELISA plates were incubated at 37 °C for 1 h. After five washing steps with PBST (300 μL/well), 100 μL of HRP-conjugated anti-M13 antibodies (with a dilution ratio of 1:5000 in PBS used in all experiments) was added to each well and then the plates were incubated at 37 °C for 2 h. After subsequent washing steps (5 times with PBS and PBST), 100 μL of 3, 3′, 5, 5′—tetramethylbenzidine (TMB) was added to each well. The reaction was then terminated by adding 100 μL of HCl (2N) to each well, and the absorbance was measured by an ELISA reader (Stat Fax 2100, Awareness Technology, Inc., United States) at 450/650 nm.

### Monoclonal phage ELISA

Based on the results of the polyclonal phage ELISA, the outputs of the round of biopanning with the highest enrichment for CD19-specific VHHs were selected for the rest of the experiments. Confirmatory colony PCR was performed on randomly selected colonies from the favorable biopanning round and the positive colonies were used for performing monoclonal phage ELISA to assess the binding capacity of each of the VHH-displaying phage particles to CD19. Briefly, each of the mentioned colonies was separately cultivated in centrifuge tubes supplemented with 5 mL LB (supplemented with 100 μg/mL ampicillin) as they were incubated at 37 °C (250 rpm) overnight. In the next step, 100 μL of the previous night’s culture was inoculated into 5 mL 2XYT culture media (supplemented with 100 μg/mL ampicillin), and the centrifuge tubes were incubated at 37 °C (250 rpm) for 2 h. After the incubation period, the tubes were supplemented with 10^9^ helper phage particles as they were incubated at 37 °C (250 rpm) for 30 min. Ultimately, each tube was supplemented with kanamycin (50 μg/mL) as they were incubated at 37 °C (250 rpm) overnight. In the following steps, the tubes were centrifuged and the phage particles in the supernatant were harvested. Next, 100 μL of the phage-containing supernatant was transferred into the wells of ELISA plates previously coated with CD19 (200 ng/well; 100 µL per well) or BSA (200 ng/well; 100 µL per well; as control). The plates were washed five times before the addition of the HRP-conjugated anti-M13 antibodies (100 µL per well). After necessary incubation and washing steps, each well was supplied with TMB (100 µL per well), after which the reaction was terminated, and the absorbance value was subsequently measured at 450 nm using an ELISA reader.

### In vitro characterization

#### Subcloning, expression, and purification of the CD19-specific VHHs

The DNA of the selected VHHs were sequenced and a set of degenerate primers were designed for their subcloning into the pET-26b(+) (Novagen; EMD Millipore) (Table 3). The reverse primer was designed in a way that a c-myc tag-encoding sequence would be introduced at the 3’ end of the VHH-encoding sequence alongside an XhoI restriction site, as the forward primer was designed to introduce an NcoI restriction site at the 5’ of the VHH sequence. The amplicons of the VHH DNA fragments were enzymatically digested with XhoI (New England Biolabs, United States) and NcoI (New England Biolabs, United States) and were ligated into the pre-digested pET-26b(+) vector using the T4 DNA ligase enzyme. The insertion of the VHH DNA fragment into the vector was validated through sequencing using the mentioned primers. The recombinant vectors were transformed into BL21 (DE3) chemically competent cells. The bacterial cells harboring the recombinant vectors were cultured in 250 mL LB medium (supplemented with 50 μg/mL kanamycin). Once an OD_600_ of 1 was obtained, isopropyl β-D-1-thiogalactopyranoside (IPTG; Thermo Fisher Scientific, United States) to a final concentration of 1 mM was added and the cultures were incubated at 18 °C (250 rpm) for 24 h. Next, the cells were harvested by a 15-min centrifugation at 12,000 ×*g* at 4 °C. The supernatant was discarded as the bacterial cells (the pellet) were washed twice with PBS. In the following step, the pellet was resuspended in lysis buffer (50 mM NaH_2_PO_4_, 300 mM NaCl, and 10 mM imidazole; pH = 8), supplemented with 1 mM phenylmethylsulfonyl fluoride (PMSF; Thermo Fisher Scientific, United States), and incubated on ice, and then they were sonicated (10 × 30 s with 30 s intervals). The solution was then centrifuged at 12,000 ×*g* at 4 °C for 30 min, and then the supernatant was collected for the purification step. Next, the solution containing the desired CD19-specific VHHs was separately loaded onto Ni–NTA columns (Sigma-Aldrich, Merck KGaA, Germany), after which the columns were washed with a wash buffer (50 mM NaH_2_PO_4_, 300 mM NaCl, and 50 mM imidazole; pH = 8) containing 50 mM imidazole to remove any non-binding proteins. Ultimately, the VHHs were eluted using different elution buffers (50 mM NaH_2_PO_4_, 300 mM NaCl, and varying imidazole concentrations including 300 mM, 400 mM, and 500 mM; pH = 8). The desalting method using dialysis membranes was taken into consideration for reducing imidazole to a negligible concentration. The purified VHHs were analyzed using SDS-PAGE. Moreover, the concentration of the purified VHHs was determined using the Bradford assay [21].

**Table 3:** Degenerate primers designed for the subcloning of the selected CD19-specific VHHs into the pET-26b(+) expression vector

Designation	Sequence
Forward-NcoI-Degenerate	5′—CATGCCATGGCCATGSAGGTSCAGCTGCWGG—′3
Reverse-XhoI-Degenerate	5′—CCGCTCGAGAAGATCTTCTTCGCTAATAAGTTTTTGTTCKGAGSWSACKGTSACC-3′

#### Determination of binding affinity

To determine the affinity constant (K_aff_) of the selected VHHs, we closely followed the ELISA-based method introduced by Beatty et al. [22]. In detail, ELISA plates were coated with different concentrations of the CD19 antigen (1.25, 2.5, and 5 µg/mL; 100 µL per well) and then they were incubated at 4 °C overnight. Next, the wells were blocked (with PBS containing 1% (w/v) skimmed milk; 100 µL per well) for 1 h at 37 °C, and after being five times washed with PBST, different concentrations (625, 1250, 2500, 5000, 10,000, and 20,000 ng/mL; 100 µL per well) of each of the selected VHHs were added to them separately. Following necessary washing and incubation steps, HRP-conjugated anti-His tag mAbs (100 µL per well; with a dilution ratio of 1:5000 in PBS used in all experiments) were added to the wells which were then incubated at 37 °C for 2 h and then washed with PBST. Ultimately, TMB (100 µL per well) was added to the wells, and following reaction termination, the absorbance was measured at 450 nm using an ELISA reader. The equation for calculating the K_aff_ as introduced by Beatty et al. and detailed below was used for further calculations [22]. In the following formula, [Ag] indicates the highest concentration of the antigen (500 ng) whereas [Ag$\mathrm{^{\prime}}$] indicates a proportion of the highest concentration of the antigen (125 or 250 ng). Moreover, [Ab] indicates the highest concentration of the antibody (VHH in this case; 1180 nM) whereas [Ab$\mathrm{^{\prime}}$] indicates a proportion of the highest concentration of the antibody (588.24, 294.12, 147.06, 73.53, or 36.76 nM).

$$\mathrm{K_{aff} }=\frac{(n-1)}{2(\mathrm{n}[\mathrm{Ab^{{\prime}}}]-[\mathrm{Ab}])}$$

$$\mathrm{n }= \frac{[\mathrm{Ag}]}{[\mathrm{Ag{^{\prime}}}]}$$

#### Binding specificity assessments

To confirm the binding specificity of the selected VHHs to CD19, we investigated whether the VHHs could bind to four different irrelevant protein molecules. For this goal, CD19, BSA, MUC1, Ovalbumin, and HER2 (5 μg/mL; 100 µL per well) were coated onto different wells of ELISA plates, and then after proper blocking, washing, and incubation steps as specified before, each of the selected VHHs (10 μg/mL; 100 µL per well) were added to the wells separately. After the addition of HRP-conjugated anti-His tag mAbs (100 µL per well) and proper incubation and washing procedures, TMB substrate was added to the wells (100 µL per well), as specified before. Following reaction termination, the absorbance of each well was measured at 450 nm.

#### Binding sensitivity assessments

To further characterize the selected VHHs, an ELISA-based sensitivity assay was conducted. To this aim, microtubes were each supplied with 50 µL (2 ng/µL; with a final concentration of 100 ng) of each of the selected VHHs separately. These VHHs were then blocked with varying concentrations of CD19 [2 ng/µL; with a final concentration of 0 ng (0 µL) to 100 ng (50 µL)] as the microtubes were incubated at room temperature for 2 h (with occasional agitation). In the meantime, the wells of an ELISA plate were coated with CD19 (100 ng per well; 100 µL per well), which were then blocked as detailed previously. In the following step, the whole mixture of each of the microtubes was added to each well and then the wells were washed five times. Next, HRP-conjugated anti-His tag mAbs (100 µL per well) were added to the wells and after necessary incubation and washing steps, the wells were supplied with TMB (100 µL per well). The absorbance of each well was measured with an ELISA reader at 450 nm, following termination of the reaction.

#### Flow cytometry analysis

To assess the binding capacity of the selected VHHs to CD19 expressed on the surface of cells, known cell lines of hematologic malignancy origin were used. In detail, K562 was considered as the CD19-negative cell line (obtained from the Iranian Biological Resource Center, Iran), and Namalwa and Raji cells were used as the CD19-positive cell lines in this experiment. The commercial anti-CD19 antibody was used as the positive control, as previously detailed. The cells were cultivated in Roswell Park Memorial Institute 1640 (RPMI 1640; Gibco, Life Technologies, United States) supplemented with fetal bovine serum (FBS; 10% (v/v); Gibco, Life Technologies, United States), and the cells were cultivated and incubated at 37 °C with 5% CO_2_. For analysis by flow cytometry, 1 × 10^6^ cells were harvested and washed twice with PBS. Next, the cells were resuspended in 100 µL PBS and the FITC-conjugated anti-CD19 antibodies (as per the manufacturer’s instructions; 5 µL per 1 × 10^6^ cells in 100 µL staining volume) were added to them for the anti-CD19 commercial antibody group in the dark, as each of the selected VHHs was also incubated separately with each of the cell lines as the rest of the experimental groups (≤ 1 µg per 1 × 10^6^ cells in 100 µL staining volume). The cells of the VHH group were washed twice, after which they were incubated with mouse anti-VHH antibodies for 2 h (1 µL which equals to 1 µg for 1 × 10^6^ cells in 200 µL PBS). After washing the excessive antibodies, FITC-conjugated goat anti-mouse IgG antibodies were added to the tubes of the VHH groups in the dark, as per the manufacturer’s instructions (0.2 µL which equals to 1 µg for 1 × 10^6^ cells in 200 µL PBS). Each experimental cell group had an unstained cell tube. Ultimately, the ability of the selected VHHs and the anti-CD19 commercial antibody to recognize and bind CD19 on the surface of the tested CD19-positive cell lines was analyzed using a Becton Dickinson FACScan flow cytometer (BD Biosciences, CA, United States).

### In silico studies

#### 3D structure prediction and assessment

The amino acid sequences of each of the selected VHHs were used as input for the process of structure prediction. In detail, the NanoBodyBuilder2 server (at https://opig.stats.ox.ac.uk) and the Robetta server (at https://robetta.bakerlab.org/) were selected as our desired 3D structure prediction servers. Of note, the favorability of the predicted 3D models by the Robetta server was determined based on their “confidence index” (from 0.0 to 1.0; 1.0 being the most favorable) and their angstrom error estimate plots (lower angstrom error estimates correspond to more favorable 3D predicted models). Furthermore, the predicted 3D model of the NanoBodyBuilder2 server and the favorable predicted model of each VHH by the Robetta server were structurally refined using the 3Drefine server (at https://3drefine.mu.hekademeia.org/) [23–25]. This server refines 3D structures by atomic-level energy minimization along with the optimization of hydrogen bond networks [23–25]. The model that showed the lowest root-mean-square deviation (RMSD) while aligned with their corresponding counterparts before energy minimization were selected for the rest of the experiments.

The 3D model of the extracellular domain of CD19 (amino acid 20–278) was also predicted by the Robetta server using the amino acid sequence of the National Center for Biotechnology Information (NCBI) reference sequence “NM_001178098.2”. This 3D model was also structurally refined, and used as the antigen (ligand) in the docking steps. All visualizations and *in silico* alignments have been performed using the PyMOL software (PyMOL Molecular Graphics System, Version 2.3.2 Schrödinger, LLC). All amino acid numberings are consistent with those of the *Kabat* numbering scheme [26].

#### Docking assessments, identification of the interactive residues, and affinity prediction

The ClusPro server (at https://cluspro.bu.edu/home.php) was utilized to carry out the docking process between each of the selected VHH and CD19 [27]. The “antibody mode” was used for this step as non-complementarity-determining regions (CDRs) were selected to be automatically masked [28]. Model selection was based upon cluster size and on energy landscape (as low energy profiles lead to larger clusters, and cluster size proportionally correlates with a higher probability of the complex) [27, 29]. Therefore, energy landscape has an indirect relationship with the most probable complex conformation [27, 29]. Moreover, the LigPlot^+^ software (version 2.2) was employed for the identification of the residue interactions between each VHH as docked to CD19 by generating two-dimensional (2D) interaction plots [30, 31]. In detail, each desired docked complex was used as the input for the software in this step. For in silico affinity prediction and predicting the impact of increasing temperature (from 25 to 37 °C) on the predicted affinity of each VHH to CD19, the PRODIGY server (at https://bianca.science.uu.nl/prodigy) was utilized [32, 33].

#### Solubility prediction

For predicting the solubility of the selected VHHs, the Protein-Sol server (at https://protein-sol.manchester.ac.uk/) was utilized. In detail, since the population average solubility (PopAvrSol) is considered 0.45, any predicted solubility (QuerySol) greater than 0.45 would be translated as being higher soluble than the average soluble *E. coli* proteins, and any scaled solubility lower than 0.45 would be indicative of a less solubility index [34, 35].

### Statistical analysis

One-way ANOVA with Tukey’s multiple comparisons test was used to determine the statistical significance (p value < 0.05) between the experimental groups. GraphPad Prism (version 9.0.1) software (GraphPad Software, Inc., CA, United States) was employed for data analyses and plot illustrations.

## Results

### Animal immunization and VHH gene library construction

**Fig. 1:**
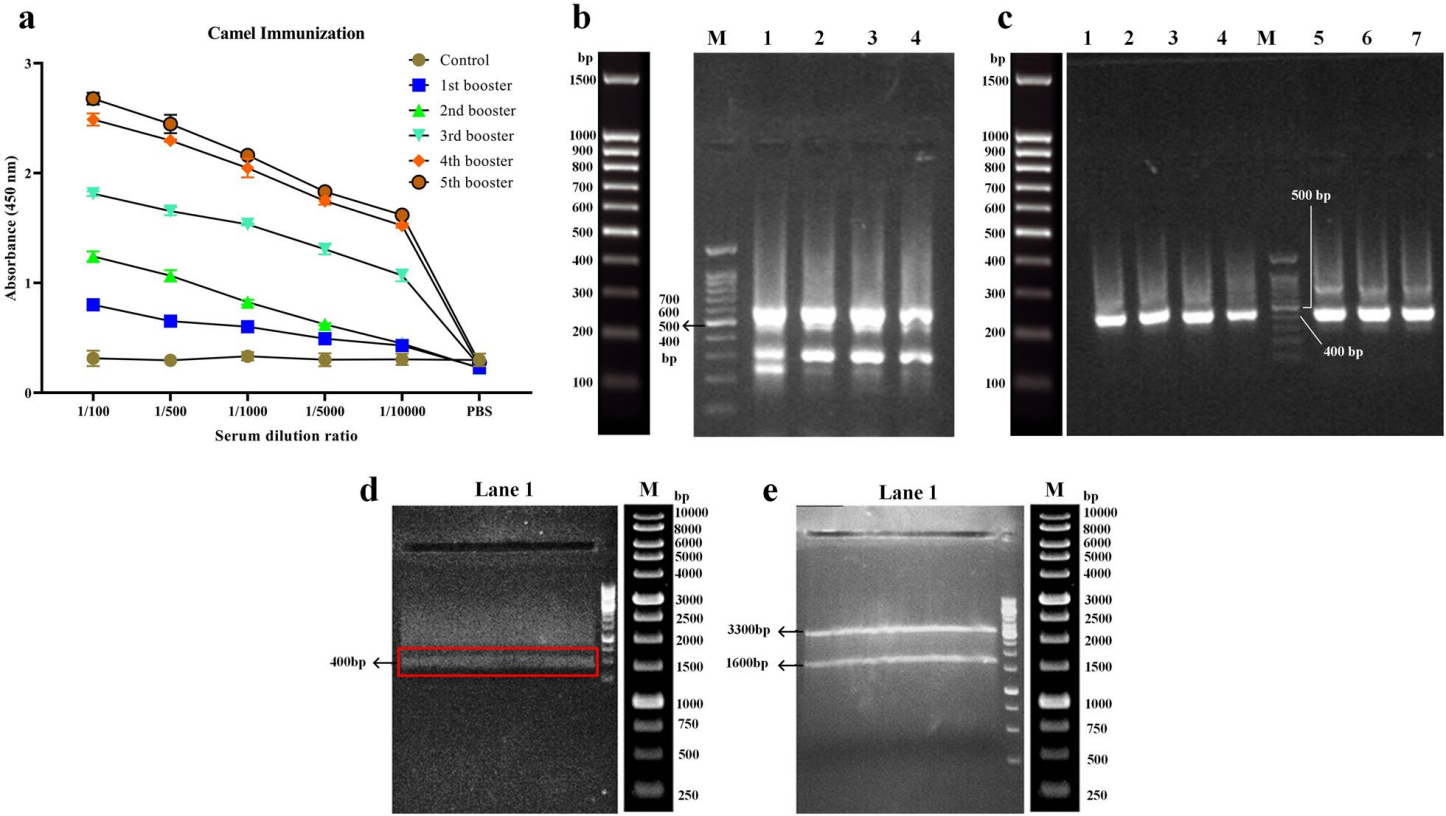
Construction of the immunized VHH library. **a**: Assessment of camel immunization by evaluating the presence of CD19-reactive antibodies in the serum of the animal using ELISA. The control consists of the animal’s serum before immunization with the CD19-positive cell lines. The values are presented as the mean of at least three replicates. **b**: Gel electrophoresis results of the first round of PCR for library construction. Lane M: DNA marker, Lane 1, 2, 3, and 4: PCR amplicons corresponding to approximately 700 bp. **c**: Gel electrophoresis results of the second round of PCR for library construction. Lane 1, 2, 3, 4, 5, 6, and 7: PCR amplicons corresponding to approximately 400 bp to 500 bp, Lane M: DNA marker. **d**: Restriction digestion of the VHH-encoding DNA fragments by SfiI. Lane 1: The SfiI-digested VHH-encoding DNA fragments, Lane M: DNA marker. **e:** Restriction digestion of the pComb3xSS phagemid by SfiI. The SfiI-digested pComb3xSS phagemid vector corresponding to approximately 3300 bp was later on extracted from the gel and used for the ligation process. Lane 1: SfiI-digested pComb3xSS phagemid, Lane M: DNA marker

An ELISA was performed on the isolated serum to assess the immune responses of the immunized camel to the injected CD19-positive cell lines. An increased immune response to CD19 was observed over time as shown by increased absorbance values for the different time points, suggesting a strong possibility of isolating CD19-specific VHHs (Fig. 1a). Following RNA extraction from the lymphocytes of the immunized camel, the cDNA was produced by RT-PCR, and then the VHH genes were amplified by the Nested PCR method. During the first round of PCR, the bands with sizes ~ 700 bp were observed that represent a specific type of antibody referred to as single-domain heavy chain antibodies (lacking CH1 domain) (Fig. 1b). The gene fragments in the range of ~ 700 bp were extracted from the gel. After gel purification, the DNA fragments were used as the template for the second round of PCR aimed at amplifying the VHH gene fragments (~ 400 to 500 bp) using specific primers designed for the regions corresponding to frameworks 1 to 4 (Fig. 1c). In the next step, the digested VHH DNA fragments and the SfiI-digested pComb3xSS phagemid vectors were ligated with the T4 DNA ligase enzyme (Fig. 1d, e, respectively). Eventually, an immunized VHH gene library was constructed with an approximate population of 6 × 10^9^. The results of the colony PCR assay on the library colonies indicated that the VHH gene is present in a very high percentage (≥ 95%) of the colonies (data not shown).

### Biopanning for the selection of CD19-specific VHHs

After isolating the library phage using the M13KO7 helper phage, five successive cycles of biopanning were carried out to obtain high-affinity VHH-displaying phages against the CD19 antigen. Moreover, the titration of the phage outputs from each cycle of the biopanning process indicated an incremental pattern in the ratio of output/input phage particles after the completion of each cycle (which itself validates the successive enrichment of CD19-reactive VHHs) (Table 4). To further corroborate the accuracy of the biopanning process, confirmatory colony PCR was performed on thirty randomly selected colonies from each biopanning round which further validated this step (data not shown).

**Table 4:** The results of the biopanning step for selecting CD19-specific VHH-displaying phages

Selection round	Input	Output	Output/input ratio	Enrichment ratio
1st	1.8 × 10^12^	1.3 × 10^7^	7 × 10^–6^	1
2nd	1.6 × 10^12^	1.8 × 10^7^	1.12 × 10^–5^	1.6
3rd	1.7 × 10^12^	6 × 10^7^	3.5 × 10^–5^	5
4th	2.6 × 10^12^	2 × 10^8^	7 × 10^–5^	10
5th	2.9 × 10^12^	4 × 10^8^	1.37 × 10^–4^	19.5

### Polyclonal phage ELISA

**Fig. 2:**
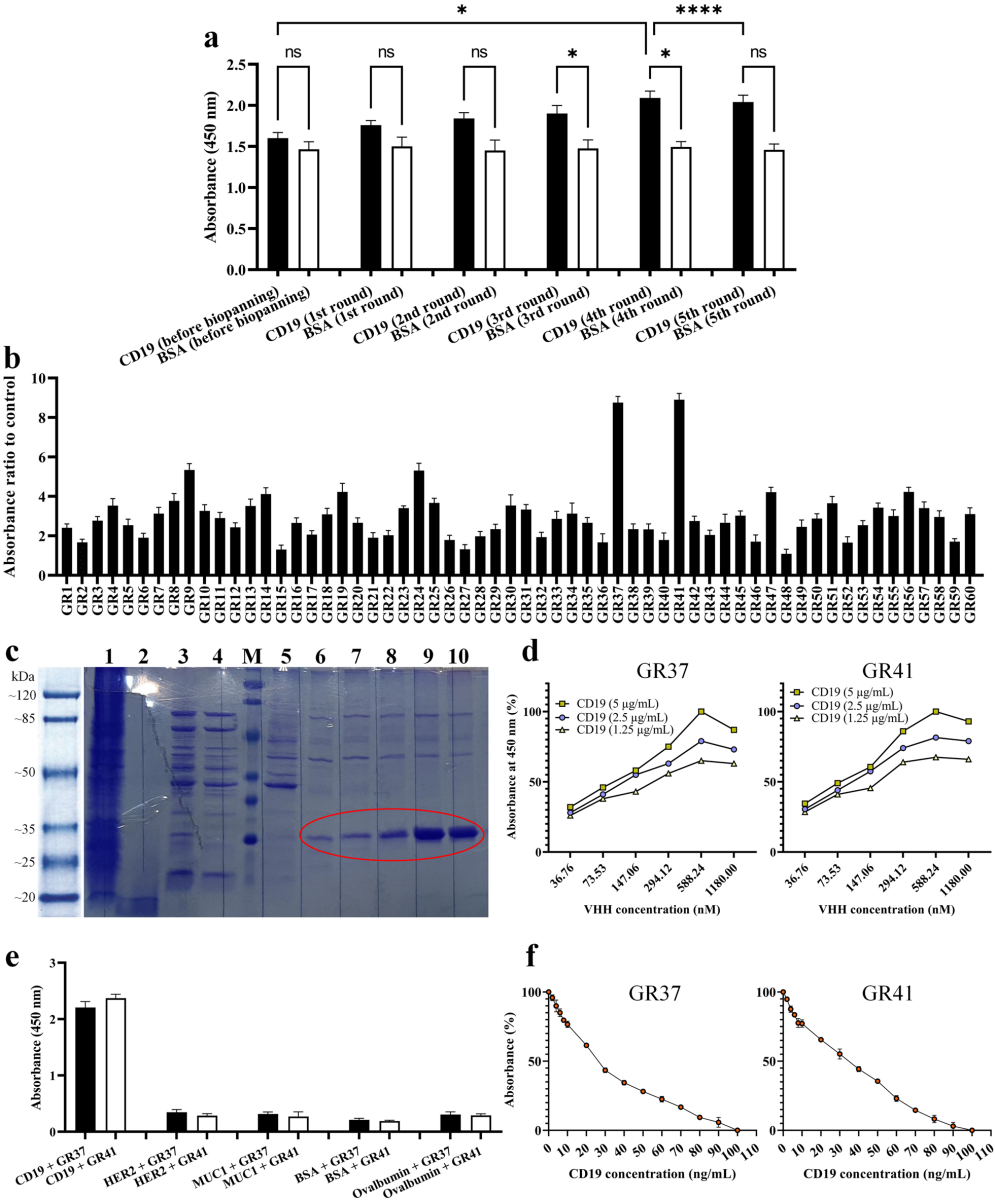
Polyclonal phage ELISA, monoclonal phage ELISA, and VHH purification and characterization. **a:** Polyclonal phage ELISA assessment of the outputs of each round of the biopanning process. The values are the mean of at least three replicates (p < 0.05). **b:** Monoclonal phage ELISA of the 4th round of biopanning. 60 random clones were selected and their binding capacity to CD19 was assessed using ELISA. The clones which exhibited a higher value of absorbance ratio to the control group (BSA) were selected for other in-depth characterization steps (GR37 and GR41). The values are presented as the mean of at least three replicates. **c:** SDS-PAGE analysis of purified GR41 expressed by pET-26b(+)-GR41-harboring BL21 (DE3) bacterial cells. Lane 1: IPTG-induced (1 mM) bacterial lysate, Lane 2: The supernatant from the culture media of IPTG-induced (1 mM) bacterial cells, Lane 3: The supernatant from the sonicated IPTG-induced (1 mM) bacterial cells, Lane 4: The flow-through fraction of IPTG-induced bacterial cell supernatant after being loaded onto a Ni–NTA chromatography column and passing through it, Lane M: protein marker, Lane 5: Column washing with 50 mM imidazole, Lane 6 and 7: Elution with 300 mM imidazole (circled red), Lane 8: Elution with 400 mM imidazole (circled red), Lane 9 and 10: Elution with 500 mM imidazole (circled red). **d**: Affinity determination of GR37 and GR41. The K_aff_ of GR37 was determined as 1.15 × 10^7^ M^−1^ whereas the K_aff_ of GR41 was calculated as 2.08 × 10^7^ M^−1^. **e**: Binding specificity of GR37 and GR41 to CD19 as determined by ELISA compared to the irrelevant proteins HER2, MUC1, BSA, and Ovalbumin. The values are the mean of at least three replicates (p < 0.05). **f**: The sensitivity profile of GR37 and GR41 to CD19. The results indicated that both VHHs are capable of detecting CD19 in a concentration as low as 2 ng/mL. The data are the mean of three replicates. * for p values < 0.05, **** for p values < 0.0001, and ns for p values > 0.05

After the biopanning process, the enriched phage library yield was appraised by performing polyclonal phage ELISA on the outputs of each round of biopanning. The phage outputs from each successive biopanning round exhibited increasing absorbance values which were relative to the successive enrichment of CD19-specific VHHs (Fig. 2a). There was no significant difference between the absorbance value of the CD19 and BSA groups in the first two rounds of biopanning; therefore, further rounds were performed. In the 3rd and 4th rounds of biopanning, the difference in the absorbance value of the CD19 and control group was statistically significant (p < 0.05). Of note, the 5th round of biopanning also yielded no significant difference between the absorbance value of the CD19 and control group. After precise analyses of the obtained results, given that the absorbance value of the 4th round of biopanning was significantly higher than that of the 5th round, the phage particles corresponding to this cycle were considered qualified for further experimental steps.

### Monoclonal phage ELISA

Of the outputs of the 4th round of the biopanning process, 100 clones were randomly selected and assessed by monoclonal phage ELISA (out of which 40 clones with the lowest absorbance ratio to the control group were eliminated). Moreover, the phage particles of each colony whose absorbance ratio to the control group was the highest were considered to be potent binders to CD19. Two clones (hereafter referred to as GR37 and GR41) had the highest absorbance ratio to the control group out of the assessed clones, and thus the highest binding capacity to CD19, and were considered for further experiments (Fig. 2b).

### In vitro characterization

#### Cloning, expression, and purification of GR37 and GR41

The DNA sequence encoding GR37 or GR41 were successfully subcloned into the pET-26b(+) expression vector (hereafter referred to as pET-26b(+)-GR37 and pET-26b(+)-GR41, respectively) as verified by DNA sequencing (data not shown). Of note, both of these VHH were expected to have a molecular weight of ~ 19 kDa (because they harbored a 2.2 kDa pelB signal sequence at their N-terminus and a C-myc tag and a 6 × His-tag at their C-terminus) while expressed in a prokaryotic host. The BL21 (DE3) bacterial cells harboring pET-26b(+)-GR37 or pET-26b(+)-GR41 were cultured and induced for protein expression as detailed previously. For the purification step, VHHs were eluted using the elution buffer containing 300 mM, 400 mM, and 500 mM imidazole with the greatest yield obtained using 500 mM imidazole. The purified proteins were assessed by SDS-PAGE, and due to the similarity in the purification step of GR37 and GR41, only the SDS-PAGE assessment of GR41 has been presented in Fig. 2c.

#### Binding affinity determination

Using the equation introduced by Beatty et al. three different K_aff_ were calculated for each of the selected VHHs corresponding to the three different CD19 concentrations that were assayed with different VHH concentrations (Fig. 2d). The final K_aff_ for each VHH was the average of three calculations. The affinity of 1.15 × 10^7^ M^−1^ (the average of 1.25 × 10^7^ M^−1^, 1.1 × 10^7^ M^−1^, and 1.1 × 10^7^ M^−1^) and 2.08 × 10^7^ M^−1^ (the average of 4.3 × 10^7^ M^−1^, 0.84 × 10^7^ M^−1^, 1.1 × 10^7^ M^−1^) was determined for GR37 and GR41, respectively (Fig. 2d). This indicates that both GR37 and GR41 are capable of strongly binding CD19.

#### Binding specificity assessment

To investigate the binding specificity of GR37 and GR41 to CD19, an ELISA-based experiment was conducted with five different antigens. The results indicated that the binding capacity of both GR37 and GR41 were significantly higher for recognizing and binding CD19, in comparison with other irrelevant target antigens (HER2, MUC1, BSA, and Ovalbumin), as indicated by significantly higher absorbance values (p < 0.05) (Fig. 2e). This corroborates that both of these VHHs are negligibly cross-reactive toward the mentioned irrelevant antigens.

#### Binding sensitivity assessment

The results of the binding sensitivity assessments were confirmatory in terms of validating the sensitivity of GR37 and GR41 to even low concentrations of CD19 (Fig. 2f). According to the results, it was elucidated that both VHHs exhibited their lowest binding capacity to coated CD19 in the groups where VHHs were blocked with 100 ng/mL of soluble antigen. Moreover, as ~ 5% of the absorbance value declined for both GR37 and GR41 in the group where VHHs were blocked with 2 ng/mL of soluble antigen, this concentration was reported as the minimum CD19 concentration detectable by the selected VHHs.

#### Flow cytometry analysis

**Fig. 3:**
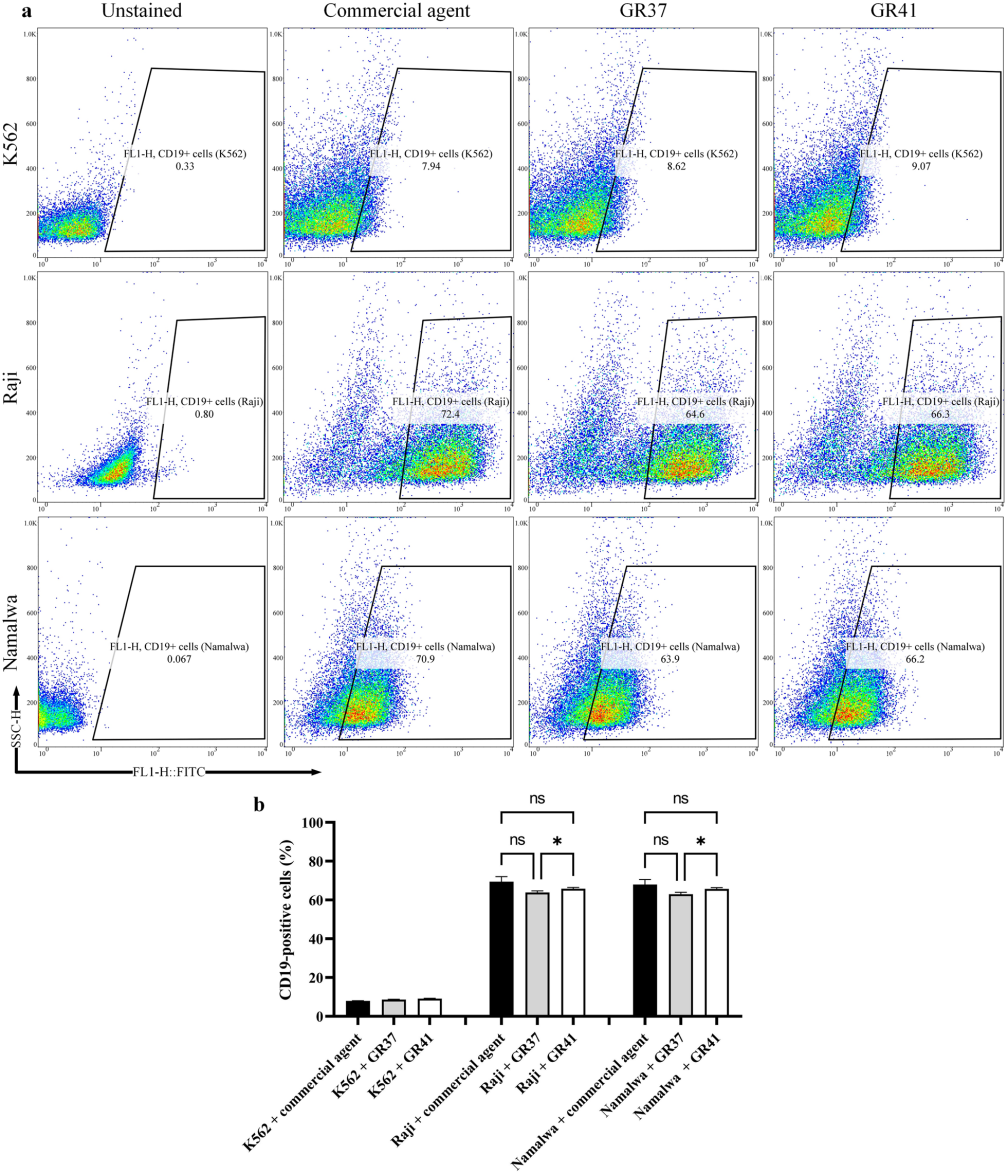
Flow cytometry dot plots for unstained, anti-CD19 commercial antibody, GR37, and GR47 in CD19-negative (K562) and CD19-positive (Raji and Namalwa) cell lines. **a**: Flow cytometry dot plots. **b:** Statistical analysis of the flow cytometric assessments. The data are the mean of three replicates (n = 3). * for p values < 0.05 and ns for p values > 0.05

The CD19-expressing cell lines of Raji and Namalwa were considered for the flow cytometry assay to further investigate the binding capability of GR37 and GR41 to CD19, in comparison with an anti-CD19 commercial antibody. In reference to the CD19-deficient cell line K562, the binding rates of the commercial antibody, GR37, and GR41 to CD19 were reported to be around 7.94, 8.62, and 9.07%, respectively. In the Raji and Namalwa cell line groups, the binding rates of the commercial antibody were 72.4 and 70.9%, respectively. Moreover, the binding rates of GR37 and GR41 were 64.6 and 66.3% in the Raji group and 63.9 and 66.2% in the Namalwa group, respectively (Fig. 3a). According to the statistical analysis (Fig. 3b), the binding capacity difference of GR37 and GR41 to CD19 expressed on the surface of Raji cells were nonsignificant in separate comparisons with the anti-CD19 commercial antibody, indicating that the binding capacity of these two VHHs is comparable to that of the commercial antibody. However, it was elucidated that GR41 significantly outperforms GR37 in terms of binding capacity to the Raji-expressed CD19 antigen. The same pattern was observed in the Namalwa group, as both of the selected VHHs bound CD19 on the surface of Namalwa cells without significant difference in comparison with the anti-CD19 commercial antibody. Also, GR41 was significantly more potent in binding CD19 expressed by the Namalwa cell line in comparison with GR37.

### In silico assessments

#### 3D structure prediction and refinement

**Fig. 4:**
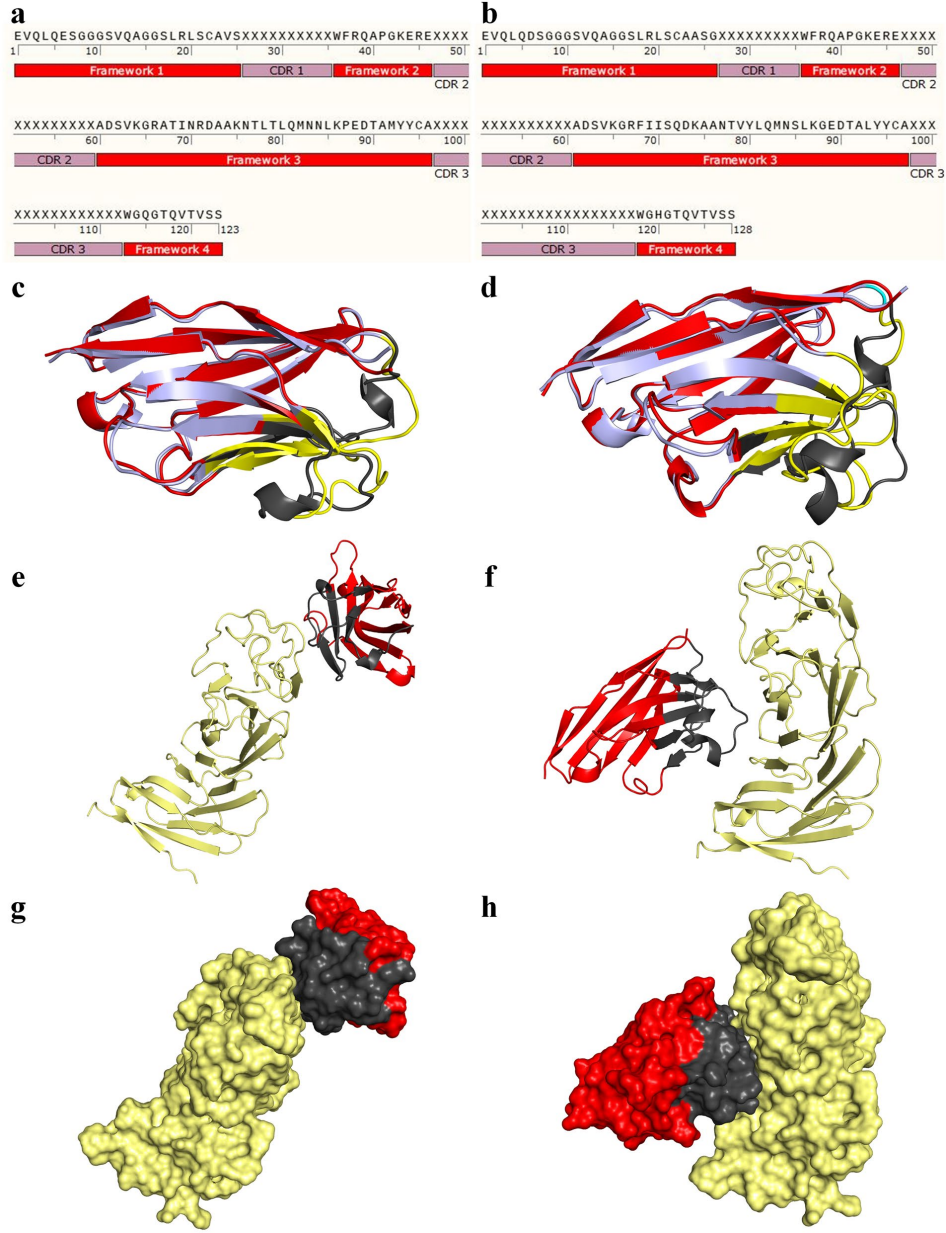
The amino acid sequences of GR37 and GR41, their predicted 3D structures, and the ClusPro results of their docking to CD19. **a** and **b:** The amino acid sequences of GR37 and GR41, respectively. **c** and **d:** The cartoon presentation of the most favorable predicted 3D model of GR37 and GR41 as modeled by the Robetta server structurally aligned with their corresponding counterparts modeled by the NanoBodyBuilder2 server, respectively. For the models predicted by the Robetta server, the framework regions are in red whereas the complementarity-determining regions (CDRs) are presented in gray. For the models predicted by the NanoBodyBuilder2 server, the framework regions are in light blue whereas the CDRs are yellow. **e** and **f**: The ClusPro results of the docking of GR37 and GR41 to CD19 in cartoon representation, respectively. The frameworks are shown in red, CDRs in grey, and CD19 in pale yellow. **g** and **h**: The ClusPro results of the docking of GR37 and GR41 to CD19 in surface model, respectively. The frameworks are shown in red, CDRs in grey, and CD19 in pale yellow

The amino acid sequence of GR37 and GR41 was obtained through their DNA sequencing and used for in-depth in silico analyses (Fig. 4a, b, respectively). The most favorable predicted 3D model of GR37 and GR41 by the Robetta server were structurally aligned with their corresponding counterpart from the NanoBodyBuilder2 server (Fig. 4c, d, respectively). An RMSD of 0.459 ångström (Å) was calculated for the 3D model of GR37 by the Robetta server aligned with its NanoBodyBuilder2 server counterpart whereas an RMSD of 0.575 was calculated for GR41. Moreover, according to the results of the 3Drefine server, the structurally refined models of GR37 and GR41 modeled by the Robetta server showed an RMSD of 0.109 and 0.104 Å while aligned with their unrefined counterparts, respectively. In reference to the NanoBodyBuilder2 server models, the refined models of GR37 and GR41 exhibited an RMSD of 0.824 and 0.796 Å while aligned with their unrefined counterparts, respectively. Based on these results, the models of the Robetta server were considered more favorable for the rest of the experiments.

#### Docking, determination of interactive residues, and the effect of temperature on predicted affinity

**Fig. 5:**
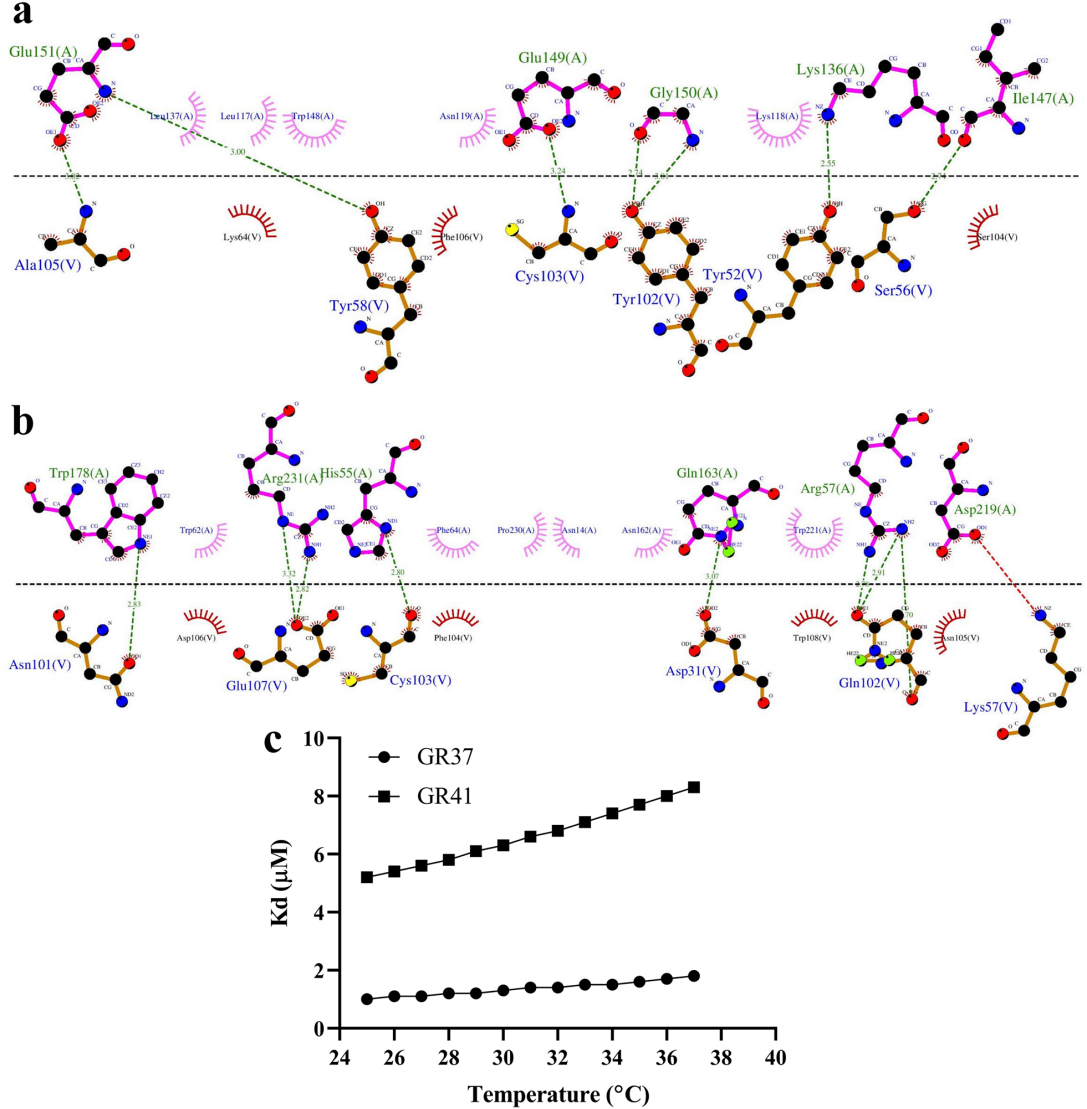
The 2D interaction plots of GR37 and GR41 as docked to CD19 and the predicted impact of temperature on their affinity to CD19. **a** and **b**: The 2D interaction plots of GR37 and GR41 as docked to CD19, respectively. The horizontal dashed line shows the interface between the VHH and the antigen. The interactive CD19 and VHH residues are represented above and below the dashed line, respectively. Hydrophobic contacts are indicated by arcs whose radiating spokes are directed toward the atoms they are in contact with. Also, dashed lines between atoms are representatives of hydrogen bonds. The VHH residues are presented with the “V” chain whereas CD19 is in the “A” chain. All antibody residue numberings are in accordance with the *Kabat* numbering scheme. **c**: The predicted impact of temperature on the predicted affinity of GR37 and GR41 to CD19

According to the results obtained from the docking ClusPro server, GR37 (Fig. 4e, g) and GR41 (Fig. 4f, h) are capable of binding CD19 at different epitopes. Furthermore, the residues involved in the interactions between each of the selected VHHs and CD19 were determined using the LigPlot^+^ software (Fig. 5a, b). Furthermore, the docking outputs of the ClusPro server were also used as inputs for affinity (ΔG) and dissociation constant (K_d_) determination using the PRODIGY server. As the temperature increased from 25 to 37 °C, both of the VHHs experienced slight degrees of affinity decline which was relatively more severe in the case of GR41 (Fig. 5c). As temperature increased from 25 to 37 °C, a K_d_ change from 1.0 to 1.8 µM was predicted for GR37 whereas a K_d_ change from 5.2 to 8.3 µM was predicted for GR41. Also, the ΔG of GR37 and GR41 bound to CD19 were predicted to be − 8.2 and − 7.2 kcal.mol^−1^, respectively, as they exhibited no fluctuations while the temperature increased from 25 to 37 °C.

#### Solubility

The Protein-Sol server predicted the solubility index of 0.519 and 0.518 for GR37 and GR41, respectively. This indicates that both VHHs might show higher solubility propensity than the average soluble *E. coli* proteins. These results are encouraging because such CD19-specific VHHs might be therapeutic agents for the treatment of hematologic malignancies; therefore, their therapeutic efficacy might be significantly dependent on their solubility index.

## Discussion

To consider immunotherapy as the future of the fight against cancer is not utterly fanciful nowadays. mAb therapy has secured its place in the market by offering a broad range of products that cover from migraine headaches [36], hemophilia [37], and severe asthma [38] to various malignancies including B-ALL, DLBCL, and CLL, as well as some solid tumors such as glioblastoma, renal cell carcinoma, and non-small-cell lung carcinoma [39]. Aside from the mentioned indications, hundreds of clinical trials are currently investigating the efficacy and safety of mAbs against a wide range of immunological and oncological conditions. Furthermore, other types of immunotherapies such as CAR-T and CAR-NK therapies have also benefited from mAbs since most of the targeting domains incorporated in CAR constructs have been derived from such mAbs [13, 14, 40]. Additionally, ADC and radionuclide therapies are also other treatment modalities that have been the beneficiaries of mAbs [41].

VHHs, known as the smallest naturally-occurring antigen-binding fragments of HCAbs, have recently been utilized for the development of therapeutics that are or will soon be under clinical investigation [14, 42]. Of note, caplacizumab (Cablivi^®^) has been the first VHH approved by the US FDA and the European Union as a combination therapy with plasma exchange and immunosuppressive agents for the treatment of adult patients suffering from acquired thrombotic thrombocytopenic purpura (aTTP) [43].

In comparison with conventional antibodies, VHHs possess peculiar characteristics that favor their wide range of applicability [42]. One of the well-known supremacies of VHHs over full-length mAbs is their slightly longer CDR3 loop which enables the targeting of hidden antigen epitopes inaccessible to conventional antibodies [16]. For instance, Koromyslova and colleagues isolated VHHs against the norovirus capsid, and demonstrated that these targeting moieties are capable of binding hidden epitope [44]. Furthermore, VHHs also exhibit a high degree of stability to temperatures alongside being highly water-soluble due to the presence of hydrophilic amino acids in their framework regions [16]. In reference to thermostability, Kunz et al. conducted an investigation on 70 VHHs and demonstrated that heat denaturation does not mediate permanent aggregation of the majority of the VHHs which were assessed [45]. Also, since VHHs share a high percentage of amino acid similarity with human antibodies, their immunogenicity index might be very low [16, 42]. However, in the cases of unfavorable immunogenicity profiles, the humanization of such VHHs might be readily carried out without any negative impact on their affinity or stability [16]. In this regard, Vincke and colleagues assessed the impact of VHH humanization on the stability, affinity, and solubility of the humanized VHH [16]. These researchers reported that substitution of residues in framework 1, 3, and 4 does not impinge on the characteristics of the VHH [16]. They also identified frameworks 2 residues that could have negative impact on the affinity of the VHH following substitution [16]. Ultimately, Vincke and co-investigators introduced a universal humanized VHH scaffold that could be leveraged for the humanization of VHHs without the loss of specificity and affinity [16].

One of the most outstanding fields in which VHHs have been applied is diagnosis through imaging [46]. Single-photon emission computed tomography (SPECT) imaging [47], near-infrared fluorescence imaging (NIR) [48], and positron emission tomography (PET) [48] are all among molecular imaging techniques that have benefited from VHHs because their low rate of unspecific tissue uptake in irrelevant organs results in high target-to-background ratio, which is in sharp contrast with that of the radio-labeled full-length mAbs. VHHs have also been applied as checkpoint blockade therapies, nanobody-drug conjugates, targeted radionuclide therapy, and VHH-based delivery systems [41, 49–51]. One of the shortcomings in the application of VHHs is their rapid renal clearance due to their small size. To address this issue, VHHs could be engineered in the format dimers (bivalent VHHs or biparatopic VHHs) or they could even be applied for the development of biparatopic HCAbs [52]. Moreover, VHH could also be genetically conjugated to toxins for the generation of immunotoxins [52].

Herein, two CD19-specific VHHs were isolated from an immune VHH gene library using the phage display technique. Our data further demonstrated that this technique is highly efficient because of its capacity for the isolation of recombinant antigen-binding fragments with strong binding affinity. Having isolated two CD19-specific VHHs, GR37 and GR41, it is our intention to apply them as the antigen-recognition domains of CAR-Ts for the development of novel VHH-based CD19-redirected CAR-T products in the future. In the context of CAR-T therapies, VHH-based CD7-redirected CAR-Ts (NCT04004637) and CD19/CD20-redirected CAR-Ts (NCT03881761) have entered clinical trials to be assessed for the treatment of certain patients T-cell ALL (T-ALL) and B-cell lymphoma, respectively [14]. All the above-mentioned examples emphasize the broad range of VHH applicability which is comparable to that of conventional antibodies. Of note, the US FDA approved Johnson & Johnson’s CAR-T therapeutic ciltacabtagene autoleucel (Carvykti^®^) for the treatment of certain groups of individuals with MM in February 2022 [14, 53]. It is encouraging to mention that this CAR-T product benefits from two sdAbs that recognize and bind distinct epitopes on BCMA [53].

Surprisingly, adoptive cell therapy has been more successful in the case of hematologic malignancies, in comparison with solid tumors. This may mainly be due to the barriers provided by tumor microenvironments (TME) to evade the tumor rejection induced by the components of the immune system [1]. From the early days of immunotherapy, various target antigens have been leveraged for the development of treatment modalities against B-cell-associated malignancies (which include CD19, CD20, CD22, CD123, ROR1, CD52, BAFF-R, CSPG4, TSLPR, and many others). Amongst these antigens, CD19 managed to gain the most attention due to numerous favorable characteristics that render it a proper cancer immunotherapy antigen. For example, in the case of ADC therapies, favorable antigens are those with adequate internalization. Comparatively, CD19 outperforms CD20 in terms of internalization; therefore, it is a preferable selection for the development of ADC-based therapies [3]. Moreover, in the case of CD20-negative disease relapse, it has been evident that CD19-based therapies can still be effective due to the maintenance of CD19 expression (4). One of the downsides of targeting CD19 is the elimination of normal B cells leading to a phenomenon called “*B-cell aplasia*” [54]. This occurrence renders the corresponding patients susceptible to opportunistic bacterial infections [54]. In the context of CAR-T therapies, accumulating evidence suggests that CAR-T products that harbor targeting domains with a moderate affinity range manage to differentiate between healthy cells (expressing the target antigen at a physiological level) and malignant cells (overexpressing the target antigen), leading to the minimization of such on-target off-tumor effects [1, 12, 55]. The affinity of our selected VHHs was determined as 1.15 × 10^7^ M^−1^ (for GR37) and 2.08 × 10^7^ M^−1^ (for GR41), which implies that CAR-Ts with these VHHs as their targeting domains might also mediate B-cell aplasia in the prospective recipients. However, this hindrance can be managed by different strategies such as reconstitution of the patient’s immunoglobulin level [54].

In the context of CD19-redirected CAR-T products, due to the systemic administration of the engineered T cells, they freely migrate to different parts of the body and enforce cytolytic reactions against cells expressing their target antigen, leading to irreversible organ damage in the case of unfavorable cross-reactivity [1]. Therefore, the cross-reactivity of the targeting domains of CAR-Ts with antigens other than the indicated one(s) is a factor of paramount importance. Herein, it was demonstrated that the selected VHHs are significantly unreactive with irrelevant antigens and they specifically recognize and bind CD19. Moreover, according to the flow cytometric analysis, the selected VHHs were also unreactive with cells deficient in the expression of CD19. Such findings might support the applicability of the selected VHHs as potential CAR targeting domains.

Various clinical findings have reported that patients undergoing CD19-redirected CAR-T therapy might, in some cases, exhibit CD19 down-regulation under treatment pressure which might leave room for possible treatment failure and disease progression [56]. Furthermore, it has been demonstrated that CAR-Ts are less sensitive in triggering cytolytic reactions against their target cells compared with endogenous T cells [40]. In detail, CAR-Ts need to engage with 100–200 target antigens via their CAR molecules to enforce cytolytic reactions compared with endogenous T cells that can be activated following the establishment of 1–10 immunological synapses with peptide-bound MHCs [40]. To overcome this limitation, CAR targeting domains need to have high affinity and sensitivity to their target antigen. According to our data, both GR37 and GR41 are highly sensitive towards CD19 rendering them as potential candidates in this matter.

Moreover, our in silico findings predicted that both GR37 and GR41 target different epitopes on the CD19 molecule (in comparison with that targeted by the CD19-specific scFv FMC63) which can increase their clinical applicability in the cases where hematologic malignancy patients experience relapse due to the resistance to a particular CD19-redirected CAR-T therapy [57, 58]. In detail, this resistance arises from mutations or alternative splicing of CD19 which results in novel CD19 isoforms no longer recognized by FMC63-equipped CAR-Ts [58, 59]. To further expand the therapeutic benefits of CD19-redirected CAR-Ts in such cases, prospective CARs need to be developed using different targeting domains. For instance, Gu and colleagues initiated a clinical study (NCT02975687) to investigate the safety and antitumor efficacy of CD19-redirected CAR-Ts engineered with the HI19α scFv as the targeting domain, rather than the FMC63 scFv, in individuals with R/R B-ALL [60]. HI19α targets a CD19 epitope which is different from that targeted by FMC63 [60]. The results from 20 patients undergoing this CAR-T treatment indicated that 18 patients (90%) achieved complete remission with incomplete count recovery (CR/CRi) in less than a month [60]. Such findings accentuate the fact that CD19-redirected CAR-T products that target CD19 epitopes distinct from that targeted by FMC63 could also be leveraged for therapeutic purposes [60]. VHHs isolated in the current study could also serve as the antigen-recognition domains of novel CD19-redirected CAR-T products. However, to identify the CD19 epitopes targeted by GR37 and GR41, future studies need to focus on epitope mapping of the extracellular domain of CD19 and crystallization of GR37 or GR41 as bound to CD19.

CD19 was selected as our target antigen in this study based on its therapeutic importance. So far, eight CD19-based immunotherapies have been in the market for medical use which accentuates the therapeutic applicability of this antigen. These commercially-available products include one ADC named loncastuximab tesirine (Zynlonta^®^) [61]. This ADC is composed of a humanized mAb conjugated to pyrrolobenzodiazepine dimer which received Orphan Drug Designation for the treatment of DLBCL and mantel cell lymphoma (MCL) [61]. Additionally, three mAbs including blinatumomab [5], inebilizumab [6], and tafasitamab [7], and four CAR-T products including tisagenlecleucel [8], axicabtagene ciloleucel [10], brexucabtagene autoleucel [9], and lisocabtagene maraleucel [11] have also been FDA-approved for medical use, targeting CD19. Moreover, targeting CD19 can be leveraged for therapeutic purposes in a variety of hematologic malignancies. For instance, Kite Pharma’s CD19-redirected CAR-T product, brexucabtagene autoleucel, has been FDA-approved for the treatment of certain patients with MCL (as the 3rd line) as well as those with B-ALL (as the 3rd line) [9, 62]. B-ALL [9], CLL [63], MCL [62], DLBCL [64], follicular lymphoma (FL) [65], small lymphocytic lymphoma (SLL) [63], Burkitt’s lymphoma [66], and even MM [67] are all among hematologic malignancies in which CD19 targeting can be leveraged for therapeutic purposes.

## Conclusion

In the current study, two CD19-specific VHHs, GR37 and GR41, with a high specificity, sensitivity, and affinity to CD19 were isolated using the phage display technique. These isolated VHHs have the potential to be incorporated as the targeting domains of VHH-based CD19-redirected CAR-Ts. Moreover, since these VHHs are derived from camelid HCAbs, their administration into humans might be subjected to elimination by neutralizing antibodies due to their immunogenicity. To address this shortcoming, future studies can focus on the humanization of the selected VHHs and an in-depth investigation of the impact of humanization on the immunogenicity, specificity, sensitivity, stability, and affinity of the VHHs.

## Data Availability

The datasets used and/or analysed during the current study are available from the corresponding author on reasonable request.

## Abbreviations

mAbs: Monoclonal antibodies
ADCs: Antibody–drug conjugates
CAR: Chimeric antigen receptor
TRBAs: T-cell-redirecting bispecific antibodies
FDA: Food and Drug Administration
NHL: Non-Hodgkin lymphoma
CLL: Chronic lymphocytic leukemia
MM: Multiple myeloma
B-ALL: B-cell acute lymphoblastic leukemia
R/R: Relapsed or refractory
NMOSD: Neuromyelitis optica spectrum disorder
DLBCL: Diffuse large B-cell lymphoma
sdAb: Single-domain antibody
HCAbs: Heavy-chain-only antibodies
FITC: Fluorescein isothiocyanate
HRP: Horseradish peroxidase
ELISA: Enzyme-linked immunosorbent assay
M-MULV: Moloney Murine Leukemia Virus-derived
*E. coli*: *Escherichia coli*
LB: Luria–Bertani
ELISA: Enzyme-linked immunosorbent assay
PBS: Phosphate-buffered saline
PBST: PBS containing 0.5% Tween
TMB: 3, 3′, 5, 5′—Tetramethylbenzidine
IPTG: Isopropyl β-D-1-thiogalactopyranoside
PMSF: Phenylmethylsulfonyl fluoride
FBS: Fetal bovine serum
CDR: Complementarity-determining region
NCBI: National Center for Biotechnology Information
2D: Two-dimensional
RMSD: Root-mean-square deviation
SPECT: Single-photon emission computed tomography
NIR: Near-infrared fluorescence imaging
PET: Positron emission tomography
T-ALL: T-cell ALL
TME: Tumor microenvironments
MCL: Mantel cell lymphoma
FL: Follicular lymphoma
SLL: Lymphocytic lymphoma
